# Higher rates of mental health screening of adolescents recorded after provider training using simulated patients in a Kenyan HIV clinic: results of a pilot study

**DOI:** 10.3389/fpubh.2023.1209525

**Published:** 2023-09-22

**Authors:** Tessa Concepcion, Peter Mogere, Kenneth Ngure, Njoroge Mwathi, Roy Njiru, Boaz Kipkorir, Catherine Kiptinness, Gakuo Maina, Emmah Owidi, Tamara Owens, Pamela Kohler, Bradley H. Wagenaar, Shannon Dorsey, Pamela Y. Collins, Jennifer Velloza

**Affiliations:** ^1^Department of Global Health, University of Washington, Seattle, WA, United States; ^2^Partners in Health and Research Development, Thika, Kenya; ^3^School of Public Health, Jomo Kenyatta University of Agriculture and Technology, Nairobi, Kenya; ^4^Simulation and Clinical Skills Center, Howard University, Washington, DC, United States; ^5^Department of Child, Family, and Population Health Nursing, Seattle, WA, United States; ^6^Department of Epidemiology, University of Washington, Seattle, WA, United States; ^7^Department of Psychology, University of Washington, Seattle, WA, United States; ^8^Department of Psychiatry, University of Washington, Seattle, WA, United States; ^9^Department of Epidemiology and Biostatistics, University of California, San Francisco, San Francisco, CA, United States

**Keywords:** HIV, global mental health, simulated patient encounters, service integration, implementation strategies, Kenya, adolescents and young adults

## Abstract

**Background:**

Kenyan adolescent girls and young women (AGYW) experience a dual burden of HIV and common mental disorders (CMD). HIV clinics are a key entry point for AGYW in need of integrated CMD and HIV care; however, rates of screening and referral for CMDs are low. Our objective was to test an evidence-based provider training strategy, simulated patient encounters (SPEs), on CMD service delivery for AGYW in a Kenyan HIV clinic.

**Methods:**

This pilot study was conducted in a public HIV clinic in Thika, Kenya from January to November 2021. The simulated patient encounter (SPE) implementation strategy included case script development from prior qualitative work, patient actor training, and a three-day SPE training including four standardized mock clinical encounters followed by quantitative surveys assessing provider competencies for each encounter. We abstracted medical record data related to HIV and CMDs such as HIV status, reason for visit, CMD screening test performed, and counselling or referral information. We conducted an interrupted time series analysis using abstracted HIV and CMD screening rates from AGYW ages 16–25 years visiting the clinic 7 months before and 3 months after SPE training. We used generalized linear models to assess changes in CMD screening rates after training.

**Results:**

A total of 10 providers participated in the training. Competency ratings improved across four mock encounters (mean score from 8.1 to 13.7) between first and fourth encounters. We abstracted all medical records (*n* = 1,154) including from 888 (76%) AGYW seeking HIV treatment, 243 (21%) seeking prevention services, and 34 (3%) seeking other services. CMD screening rates increased immediately following training from 8 to 21% [relative risk (RR) = 2.57, 95% confidence interval (CI) = 1.34–4.90, *p* < 0.01]. The 3 months following the SPE training resulted in an 11% relative increase in CMD screening proportion compared to the 7 months pre-SPE (RR: 1.11, 95% CI: 1.04–1.17, p < 0.01). Finally, 1% of all pre-SPE screens resulted in referral versus 5% of post-SPE screens (*p* = 0.07).

**Conclusion:**

The SPE model is a promising implementation strategy for improving HIV provider competencies and CMD service delivery for adolescents in HIV clinics. Future research is needed to explore effects on adolescent clinical outcomes in larger trials.

## 1. Introduction

Common mental disorders (CMDs), including depression, anxiety, substance abuse, and post-traumatic stress, are especially prevalent among adolescents and often co-occur with HIV risk and related morbidity and mortality (1). Recent studies conducted in Kenya and other sub-Saharan African countries found a 30%–50% prevalence of CMDs among adolescent girls and young (AGYW) between the ages of 16–25 years who were seeking HIV treatment or prevention services (2–6). CMDs can increase risk for or exacerbate symptoms associated with HIV. For example, poor mental health is associated with reduced adherence to HIV-related medications and an HIV diagnosis can contribute to depression and anxiety (2–4, 7–9). In addition, CMDs and HIV share social and structural determinants of risk (e.g., gender-based violence, food insecurity) that contribute to the dual burden of disease among AGYW (1).

Integrating CMD screening, referrals, and evidence-based treatments with HIV prevention and treatment programs has the potential to address both mental and physical needs of AGYW (1). The World Health Organization (WHO) recommends routine CMD screening at HIV service delivery points to synergistically improve mental health and HIV outcomes in settings with high burden of both conditions including Kenya (6, 10–13). Systematic reviews on HIV and mental health service integration have emphasized that successful care integration requires training HIV providers in CMD symptom identification, basic adolescent-friendly counseling skills, and referral pathways (14, 15). AGYW at HIV clinics in Kenya have expressed the need for integrated mental health and HIV services, but also have concerns about HIV providers’ negative attitudes about mental health and a lack of recognition of mental health needs (16). HIV providers themselves have also expressed hesitancy with offering routine CMD screening for HIV clinic patients, citing lack of confidence, competency, and training to provide mental health services (17).

Strategies are needed to enhance HIV provider competencies around mental health service delivery to reduce the mental health treatment gap and improve patient-centered care for AGYW in Kenya (18, 19). Simulated patient encounters (SPE) are an implementation strategy that involve training standardized patient actors (SPs) to work with healthcare providers in mock clinical encounters for training and evaluation (20). This approach has been shown to improve provider skills in clinical assessment, patient-centered communication, and counseling (21). SPE training uses case scripts representing typical service users, provider training didactics, role-plays, competency assessments, and feedback models to improve clinical outcomes. In Kenya, simulated patients have been used to improve PrEP service delivery for AGYW, quality of care for childhood illnesses, quality of sexually transmitted disease case management, and adolescent retention in HIV care (22–26). Within this growing body of evidence, the SPE approach has not yet been tested in Kenya to improve CMD care delivery among AGYW receiving HIV services. The primary objective of this pilot study was to test the effect of an SPE training on mental health service delivery for Kenyan AGYW in HIV clinic settings. We hypothesized that an SPE strategy could improve HIV provider competencies to deliver mental health screening and referral for AGYW in a Kenyan HIV clinic, which could in turn improve mental health and HIV prevention and treatment outcomes among AGYW (27).

## 2. Materials and methods

### 2.1. Study design and participation

This work was conducted in three phases: (1) qualitative research to inform SPE strategy development; (2) SPE training and provider competency assessments; and (3) an interrupted time series analysis assessing the effect of the SPE training on CMD screening rates among AGYW attending a Kenyan HIV clinic. This study was conducted with providers (phases 1 and 2) and patients (phases 1 and 3) in Thika, Kenya at Thika sub-County Hospital, a public facility providing HIV prevention [HIV testing, pre-exposure prophylaxis (PrEP)] and treatment [antiretroviral therapy (ART)] services to Kenyan AGYW. The study took place from January 2021 to November 2021.

### 2.2. Phase 1: intervention development

#### 2.2.1. Qualitative data collection and analysis

We conducted a qualitative exploratory study among AGYW and HIV and mental health providers which informed the development of four standardized patient case scripts and provider competency checklists for the SPE training (28). Qualitative data from this phase were analyzed only for the purpose of developing case scripts. The methods and results from this phase have been previously described (28).

### 2.3. Phase 2: SPE training

Six professional Kenyan actors with previous experience conducting role plays for HIV outreach events were trained to portray simulated patients as depicted in our case scripts. The case scripts represented AGYW seeking HIV services and presenting a range of symptoms of CMDs (Supplementary File 1). All actors were young women, ages 18–25, and were trained in the standardized patient methodology over 3 days on Zoom by an expert trainer. The actor training curriculum followed the Association of Standardized Patient Educators (ASPE) Standards of Best Practice (29). Also because the training was virtual, the training curriculum incorporated aspects of a multimodal model for online education which integrates relevant theoretical frameworks for online education (30). The model emphasizes the concept of learning communities which enhanced the actor’s knowledge acquisition, active engagement, and overall experience. The training curriculum included the three areas: (1) fundamentals of simulated patient methodology, (2) case portrayal, and (3) delivering constructive feedback. The first section was a didactic lecture to educate actors on the fundamentals of being an authentic realistic patient for the provider training. The next area focused on training actors on how to assume the character embedded in the case scripts. One actor was assigned to each case script for the provider training. Each case script had secondary actors trained to perform the case if needed (e.g., due to an unexpected illness or absence among another actor). The third training area was on delivering case specific constructive verbal feedback to the providers. The feedback training emphasized specific points; each actor should focus on based on their case script. Since there were multiple actors portraying the same case script and delivering subsequent feedback, it was important to establish consistency, standardization, and accuracy of the portrayal and feedback delivery. Inter-performance reliability and consistency was established with actor active engagement in role-plays, paired practice, and small group discussions. These activities allowed the actors to practice and receive feedback on their case portrayal and feedback delivery. Actors had the opportunity to be the performer and receive individual feedback as well as learn through the observation of their peers performing. The actors were encouraged to ask questions and self-reflect on how to improve. At the end of the training workshop, all actors participated in a “dry run” assessment to confirm their readiness to participate in the provider training. The trainer and the study team rated each actor using an evaluation form. The evaluation form consisted of six questions with a nominal yes/no scale for each question. The questions assessed the actor’s readiness to proceed to the provider training. Actors had to receive all yes scores from both the trainer and study team member to move forward. Any actor who did not fall into this category were considered for re-training or dismissal from the study.

We then conducted an in-person three-day SPE training with 10 providers at the Thika sub-County Hospital. Eligible providers included those who were currently a doctor, clinical provider, or nurse providing HIV prevention or treatment services to AGYW (such as routine HIV testing, and PrEP delivery for HIV prevention, or ART delivery for HIV treatment). All eligible providers were invited to participate in the SPE training. SPE training materials were developed based on qualitative findings, relevant literature on CMD screening, counseling, and treatment, and Kenyan Ministry of Health guidelines on mental health and HIV service provision. Day 1 included didactic content on CMD symptom presentation, screening tools, and active referral techniques. We discussed adolescent-friendly communication skills (e.g., protecting confidentiality, non-judgmental and warm tone). Didactic content on screening tools was limited to existing tools available at the clinic; no new CMD screening tools were introduced to the trainees. Staff were trained in English versions of tools. Days 2–3 of the training included mock clinic encounters with the standardized patient actors. Each provider completed four mock encounters with the SPs to be exposed to each of the four case types. After each encounter, actors provided verbal feedback directly to the provider, describing how they felt during the encounter, things the provider did well, and areas for improvement related to adolescent-friendly communication and elicitation of CMD symptoms. All patient encounters were video recorded for training purposes.

#### 2.3.1. Provider competence measures

After each encounter, the patient actors, other healthcare providers, and research team members evaluated each provider’s skills in adolescent-friendly communication, assessment, and screening for CMDs, and any provision of referral services or basic counseling to triage the patient as needed. Evaluations were conducted using standardized competency checklists (Supplementary File 2; total possible score was between 0–15) with the following domains: assessed CMD symptoms via screening (possible score: 0–3); assessed next clinical care steps based on screening results (possible score: 0–3); made appropriate referral(s) as needed (possible score: 0–3); displayed active listening skills (possible score: 0–3); and asked clarifying questions (possible score: 0–3). Score level 0 reflected that the domain was not done, level 1 that it was completed vaguely or poorly, level 2 that it was partially completed, and level 3 that it was completed fully. Competency scales were developed based on the literature and goals of our case scripts and SPE training materials. The study team discussed the scales to establish content validity. Scales were also reviewed by the team and the patient actors during their training to discuss and troubleshoot potential quality control issues with their completion.

Healthcare providers completed surveys before and after the SPE training to assess knowledge and self-rated competency in mental health service delivery (screening, participant triage as needed, providing referrals accurately and with warm handoffs). Surveys included a 12-item knowledge questionnaire with Likert responses to questions like, “People with depression or anxiety could snap out of it if they wanted.” The scales were developed from prior work with women in Kenya (31). Items were scored from 0–4 and summed (possible range: 0–48), with a higher total score indicating greater knowledge around CMDs. Providers also completed an 11-item questionnaire related to their self-rated competencies for mental health service delivery with Likert responses to questions such as, “I feel confident in my ability to screen AGYW for common mental disorders like depression, anxiety, and stress.” Items were also scored from 0–4 and summed (possible range: 0–44), with a higher score indicating greater self-rated competency for delivering mental health services to AGYW in HIV care settings.

We used descriptive statistics to explore self-rated provider knowledge and competencies before and after the training. We also assessed average provider competency ratings across the four clinic encounters to quantify any improvement in competencies over time.

### 2.4. Phase 3: interrupted time series analysis

We conducted an interrupted time series study design to assess changes in CMD screening rates among AGYW seeking routine care after the SPE intervention. Our primary outcome was the proportion of clinic visits with a documented CMD screening. Secondary outcomes included type of CMD screening performed, proportion of visits that included brief counselling and triage of active CMD symptoms, and proportion of visits that resulted in referral.

#### 2.4.1. Clinical record data abstraction

Two staff members abstracted clinical medical record data from Thika sub-County Hospital from January 4–November 26, 2021. The SPE training occurred over 3 days from August 21–24, 2021. For this analysis, the pre-SPE period is from January 4–August 21, 2021. The post-SPE period is from August 25–November 26, 2021. Data were abstracted for all clinic visits among AGYW who were: ages 16–25 years, female at birth, and currently seeking HIV prevention or treatment services, including HIV testing, PrEP, or ART. We developed and piloted our abstracted form to ensure data availability in the hospital records, including both paper-based and electronic charts. Abstracted data included: identification number (to link records for AGYW with multiple clinic visits during the study period); demographics (i.e., age, relationship status); HIV status and services sought (e.g., reason for clinic visit, HIV status, length of time receiving HIV services at that clinic, and medication adherence); and mental health services provided (i.e., screening, counselling, and referrals). Chart notes included information on whether any CMD screening was conducted at a visit, the type of CMD screened for, screening tool used, and the result of the screening. They also included open text fields describing the quality and nature of any counseling conducted and referrals provided. Counselling was defined as any counselling topic related to CMDs (e.g., depressive symptoms, gender-based violence, trauma). Any screening tool related to CMD was included in the abstraction. Medical records may have reported that a screening for a particular CMD was performed but did not include the tool used. Providers at the clinic were trained in and had access to one screening tool alcohol use [CAGE-AID/CRAFFT (32, 33)], one for depressive symptoms [PHQ-9 (34)], one for general mental health [SRQ-20 (35)] and one for posttraumatic stress symptoms [“Post Rape Care” (PRC) form (36)]. Alcohol use and PRC forms were included in electronic medical records (EMR) while depressive symptom and general mental health tools were completed by hand and kept in the patient’s physical file. In cases where abstracted records included a CMD screening tool score, we used the following cutoffs: the SRQ-20 screening results used a depressive symptom cutoff of 7 (35) and the PHQ-9 used depressive symptom cutoffs of 5–9, 10–14, 15–19, and 20–27 to indicate mild, moderate, moderately severe, and severe levels of depressive symptoms, respectively (34).

#### 2.4.2. Statistical analysis

We used descriptive statistics (frequencies, medians) to summarize demographic information, CMD service outcomes, and HIV-related outcomes. We used an interrupted time series design to compare CMD screening rates before and after the introduction of our SPE training. CMD screening rates were calculated by dividing the number of clinic visits with any CMD screening conducted by the total number of clinic visits each week (aggregated by week to account for daily fluctuations in clinic volume). We used scatterplots to identify the underlying trend in CMD screening, any seasonal patterns in CMD screening and clinic visit frequency, and outliers. Models were fit using generalized linear regressions with log link, Poisson distributions, and robust standard errors. Exposure to the SPE training was coded as a binary variable for the pre/post SPE periods.

We assessed autocorrelation of the interrupted time series through partial autocorrelation function (PACF) plots and in cases where we found meaningful and significant autoregression we conducted a sensitivity analysis using an autoregressive integrated moving average (ARIMA) model. We also performed several additional sensitivity analyses, including: (1) aggregating CMD screening rates by day to consider more granular changes in CMD screening proportion; (2) aggregating CMD screening rates by month to account for monthly fluctuations in CMD screening proportions and clinic volume; and (3) truncating data in the week immediately after the SPE intervention to account for potential Hawthorne effect (alteration of study subject behavior due to awareness of being observed) (37).

Due to the small number of clinic visits with any counselling or referrals conducted, we descriptively summarized the proportion of clinic visits with CMD counseling or referrals provided, among clients who had any CMD screening conducted. Chi-square statistics were used to quantify any statistically significant differences in proportion of visits results in counseling and referral between the pre-SPE and post-SPE periods.

### 2.5. Ethical considerations

Institutional review boards at the University of Washington and Kenya Medical Research Institute Scientific and Ethics Review Unit (KEMRI SERU) approved this study. All participants provided written informed consent in English or Kiswahili before participation in the in-depth interviews and SPE training in phases 1 and 2. AGYW were not consented for medical record abstraction in phase 3. We received a written agreement from the Thika sub-County Hospital prior to beginning data abstraction and only abstracted de-identified data.

## 3. Results

### 3.1. Provider competence

Provider competency scores increased across the four mock encounters as indicated by the average rating on the standardized competency checklists. Specifically, the 10 providers had a mean competency score of 8.1 [standard deviation (SD): 1.1] for their first simulated patient encounter compared with a mean score of 13.7 (SD: 1.7) for their fourth simulated patient encounter (*p*-value <0.001). The largest improvement in scores was between the first and third case (average of 3.1-point difference in mean score between the first and third cases), whereas there was a smaller improvement observed between the third and fourth cases (average of 1.6-point difference in mean competency score between these cases). Checklist sub-scores also improved across the four cases for both the CMD-specific and interpersonal competencies in the checklist. We observed a statistically significant improvement in the mean score for three items around screening for CMDs, accurately assessing next steps in care based on screening results and making appropriate referrals (from 3.1 on the first encounter to 7.9 on the fourth encounter, *p* = 0.02). We also found statistically significant improvements in mean score for two items around adolescent-friendly communication skills of active listening and asking clarifying questions (from 1.6 on the first encounter to 5.8 on the fourth encounter, *p*-value = 0.01).

Mean scores around provider knowledge and self-rated competencies for mental health service delivery significantly increased from before the SPE training to the end of the training. Providers had a mean knowledge score of 26.1 (SD: 3.2) prior to the training and 37.8 (SD: 4.7) after the training (*p*-value: 0.01). Their mean reported self-efficacy score was 29.8 (SD: 3.6) prior to the training and 38.5 (SD: 5.1) after the training (*p*-value: 0.02).

### 3.2. Pilot findings on SPE effectiveness for CMD screening

A total of 1,154 medical records abstracted, of which 769 (66.7%) were from the pre-SPE training period and 385 (33.3%) were from the post-SPE training period. Throughout the study, 528 individual women were seen at the clinic; Most had only one visit during this study (*n* = 310, 58.7%), 55 had 2 visits (10.4%), 42 had 3 visits (8.0%), and 121 had 4 or more visit (22.9%). The median age of AGYW seeking care at Thika sub-County Hospital in this time frame was 21 years [interquartile range (IQR): 19–23; Table 1]. Based on abstracted data throughout the study, approximately 28% of AGYW seeking HIV services in the clinic were in school (*N* = 314) and most were single with no partner (*n* = 672, 58.23%). The proportion of AGYW who were living with HIV and seeking care at the clinic differed between the pre- and post-SPE periods (79.06% vs. 72.73%, respectively; *p* = 0.02). Specifically, compared to the pre-SPE period, fewer AGYW had clinic visits for HIV treatment (72.21% vs. 77.89%, *p* < 0.01) and more attended the clinic for other reasons such as GBV counselling (6.75% vs. 1.04%, *p* < 0.01).

**Table 1:** Demographic and HIV service outcome among AGYW seeking care at Thika sub-County Hospital between January and November 2021.

	Total (*N* = 1,154)	Pre-SPE (*N* = 769)	Post-SPE (*N* = 385)	*p*
**Age** (median, IQR)	21 (19–23)	21 (19–23)	22 (19–24)	0.022
**Number of school years completed** (median, IQR)	14 (12–14)	14 (12–14)	13.5 (12–14)	0.290
Missing	866 (75.04%)	565 (73.47%)	301 (78.18%)	
**Job/occupation**				0.110
Student	314 (27.21%)	214 (27.83%)	100 (25.97%)	
Missing	700 (60.66%)	451 (58.65%)	249 (64.68%)	
**Current marital status**				0.568
Single, no partner	672 (58.23%)	461 (59.95%)	211 (54.81%)	
Single with partner	142 (12.31%)	94 (12.22%)	48 (12.47%)	
Married (husband has one wife)	184 (15.94%)	119 (15.47%)	65 (16.88%)	
Widowed	8 (0.69%)	5 (0.65%)	3 (0.78%)	
Divorced/separated	19 (1.65%)	12 (1.56%)	7 (1.82%)	
Missing	129 (11.18%)	78 (10.14%)	51 (13.25%)	
**Earns own income**	118 (10.23%)	84 (10.92%)	34 (8.83%)	0.104
Missing	452 (65.04%)	199 (63.38%)	253 (66.4%)	
**HIV status**				0.016
Positive	888 (76.95%)	608 (79.06%)	280 (72.73%)	
Negative	266 (23.05%)	161 (20.94%)	105 (27.27%)	
**Reason for clinic visit**				<0.001
HIV treatment	877 (76.00%)	599 (77.89%)	278 (72.21%)	
HIV testing or prevention (e.g., PrEP)	243 (21.06%)	162 (21.07%)	81 (21.04%)	
Other^a^	34 (2.95%)	8 (1.04%)	26 (6.75%)	
**Months receiving services at clinic**^b^ (median, IQR)	60 (12–144)	72 (12–144)	60 (10–144)	0.352
Missing	201 (17.42%)	102 (13.26%)	99 (25.71%)	
**Morisky medication adherence score**				
Good	100 (8.67%)	77 (10.01%)	23 (5.97%)	0.123
Inadequate	12 (1.04%)	9 (1.17%)	3 (0.78%)	
Poor	9 (0.78%)	6 (0.78%)	3 (0.78%)	
Not completed	1,033 (89.51%)	677 (88.04%)	356 (92.47%)	0.065
Missing	7 (0.61%)	5 (0.65%)	2 (0.52%)	

a Other responses included: GBV (*n* = 33), counselling services (*n* = 1).

b Among AGYW living with HIV.

**Figure 1:**
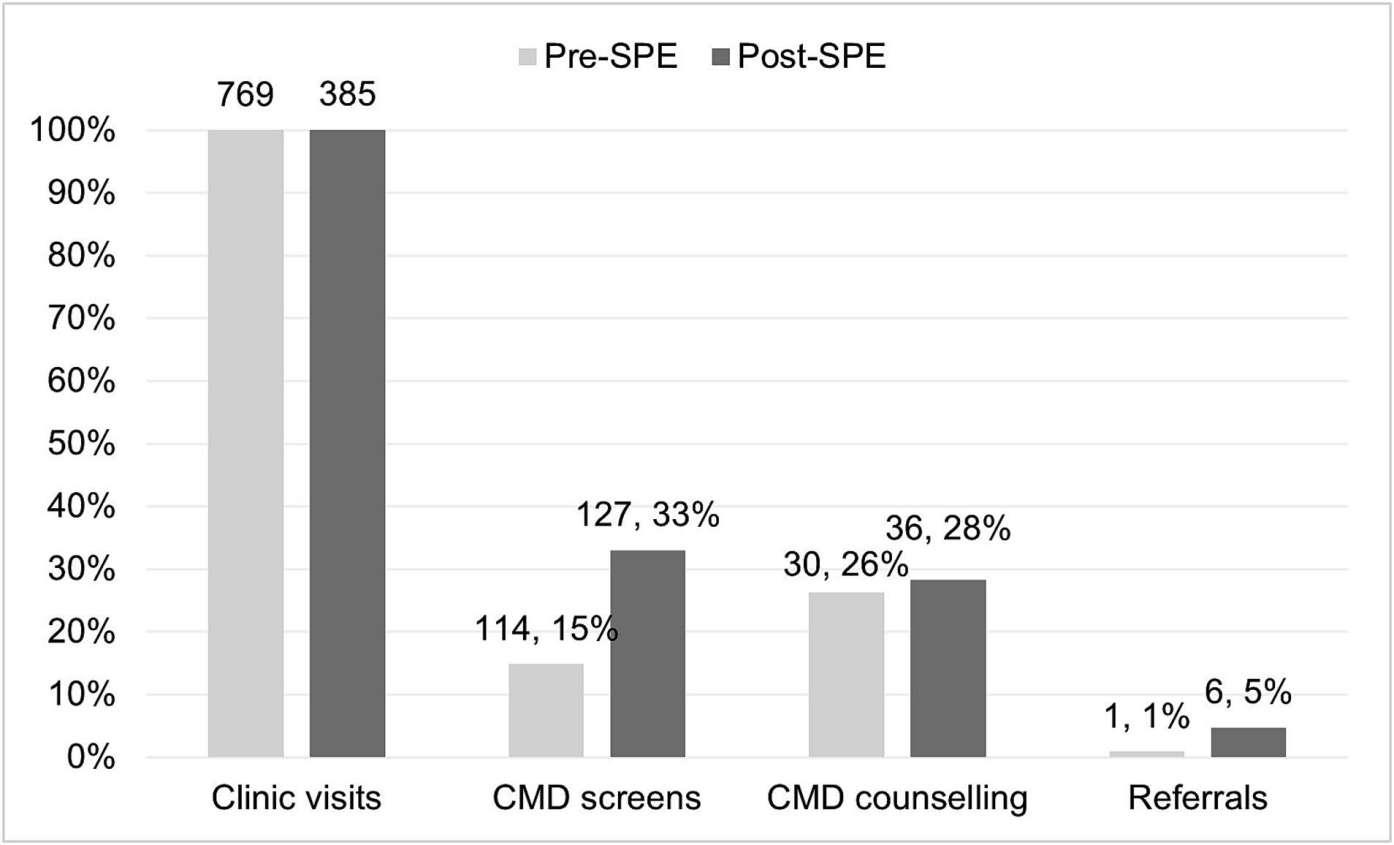
Common mental disorder (CMD) treatment cascade^*^. ^*^CMD screens represented as proportion of clinic visits; CMD counselling and referrals represented as proportion of visits with a CMD screen.

In the pre-SPE period, 114 of 769 (15%) of clinic visits included screening for CMDs, whereas, in the post-SPE period, 127 of 385 (33%) of clinic visits had a CMD screen (*p* < 0.01) (Figure 1). In both the pre- and post-SPE periods, substance use was the most CMD screened for (11.1% of visits pre-SPE, 22.6% of visits post-SPE). No visits had a screening completed for depression or anxiety in the pre-SPE period compared to 19 visits (4.9%) post-SPE training. The most common screening tool reported in medical records was the PRC form (3% of visits pre-SPE, 6% of visits post-SPE), followed by CAGE-AID/CRAFFT (1% of visits pre-SPE, 5% of visits post-SPE), SRQ-20 (0% of visits pre-SPE, 4% of visits post-SPE), and PHQ-9 (0% of visits pre-SPE, 2% of visits post-SPE). In visits where the SRQ-20 was completed, five AGYW had an elevated symptoms score. In visits where the PHQ-9 was completed, two AGYW had mild depressive symptoms, one had moderately severe depressive symptoms, and one had severe depressive symptoms.

**Figure 2:**
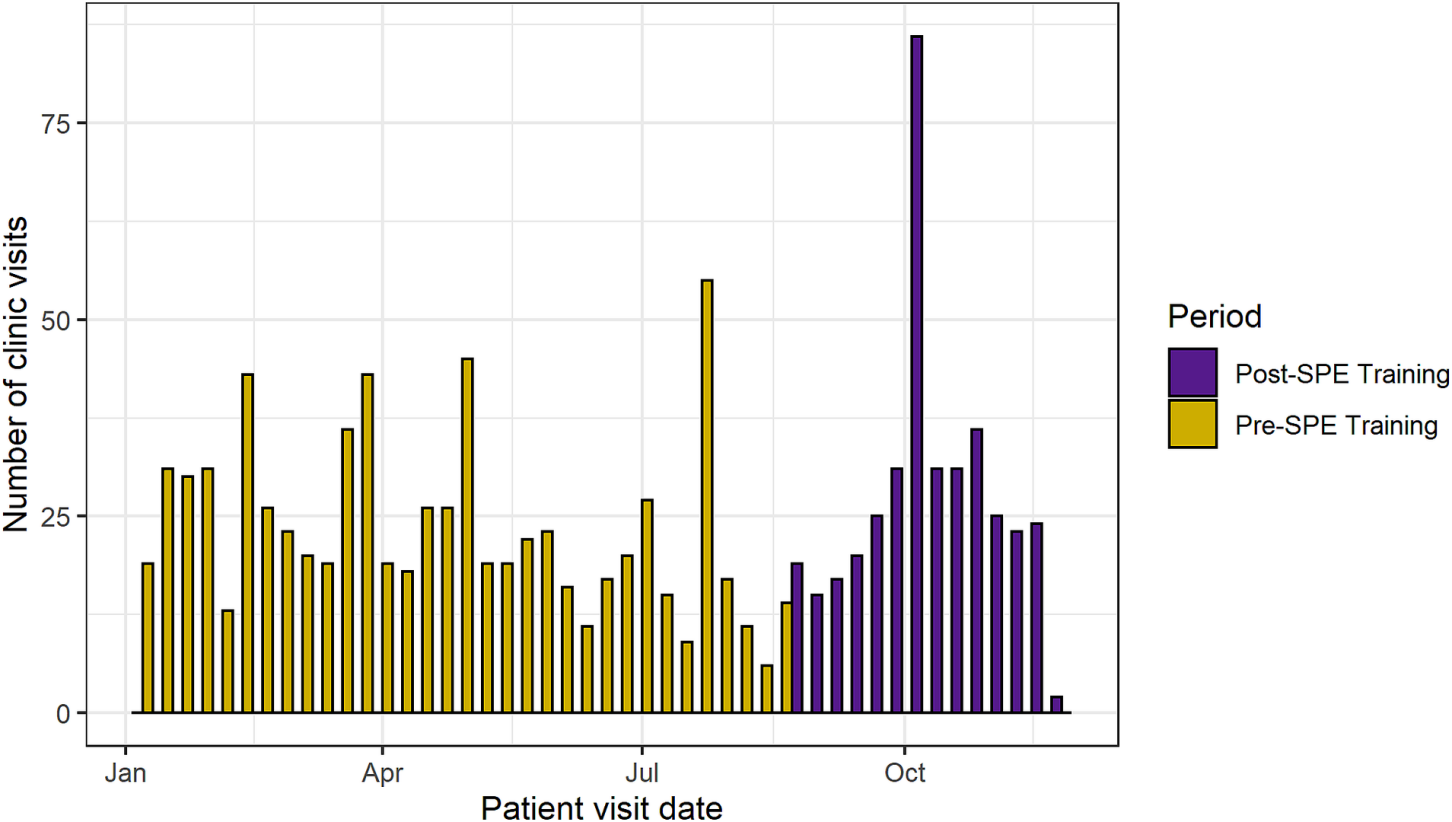
Total number of clinic visits by week.

**Figure 3:**
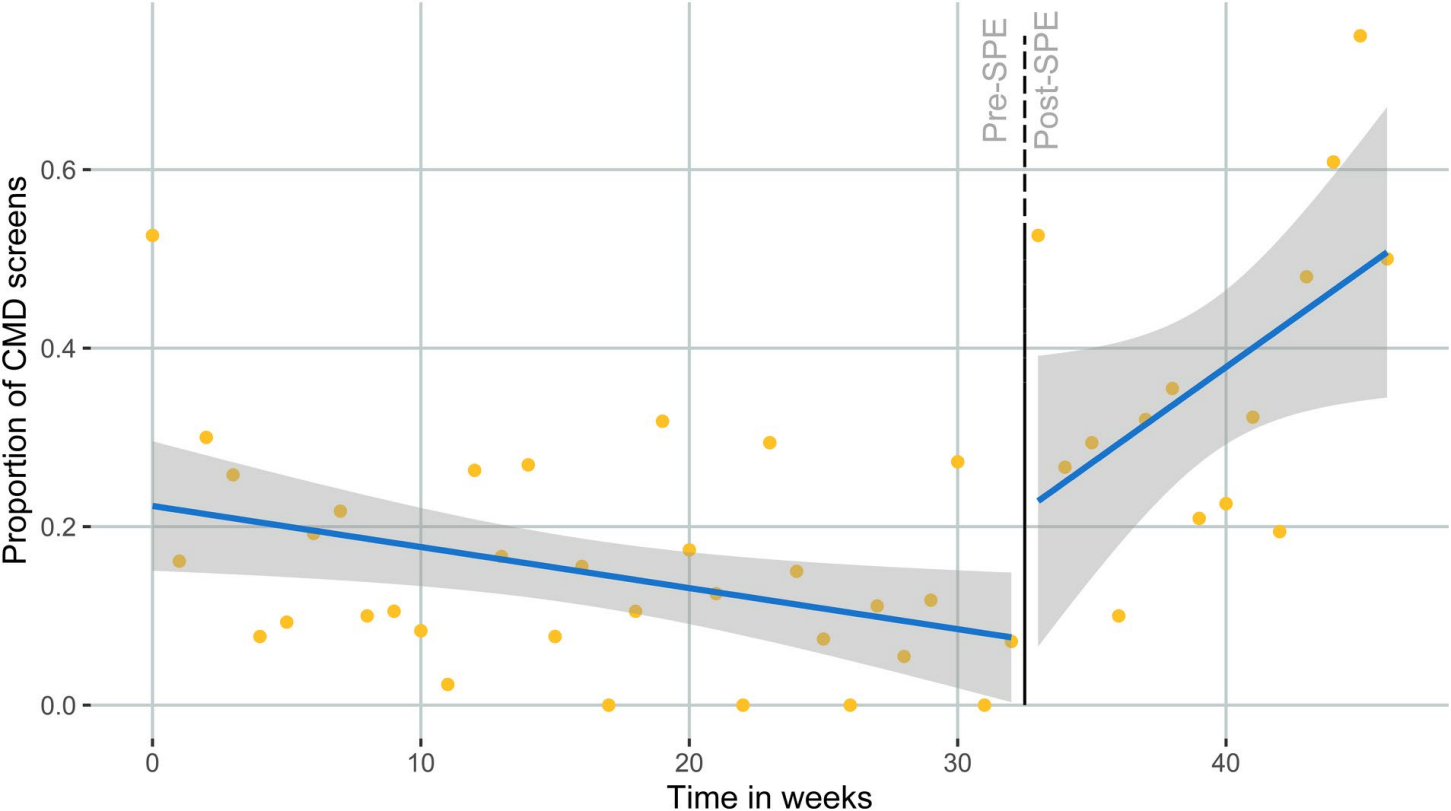
Proportion of clinic visits with a CMD screen, before and after SPE training.

The median number of AGYW clinic visits per week was 22 (IQR: 17–31) (Figure 2). There was no significant difference in the number of clinic visits per week between the pre- and post-SPE periods (pre-SPE median = 20 visits, IQR: 17–27; post-SPE median = 24.5 visits, IQR: 19–31; *p* = 0.18). The estimated proportion of CMD screens at baseline (first week of medical record abstraction—January 2021) was 22% (Table 2). We found a statistically significant 2.6-fold higher proportion of AGYW screened for CMDs the week immediately after SPE training for providers (RR: 2.57, 95% CI: 1.34–4.90, *p* < 0.01). The estimated proportion of CMD screens went from 8.3% the week before the SPE training to 20.5% the week after the SPE training. Prior to SPE training, there was an estimated 3% decrease in CMD screens among AGYW each week (RR: 0.97, 95% CI: 0.95–0.99, *p* < 0.01). The 3 months following the SPE training resulted in an 11% relative increase in CMD screening proportion compared to the 7 months pre-SPE (RR: 1.11, 95% CI: 1.04–1.17, *p* < 0.01) (Figure 3). Sensitivity analyses showed similar effect sizes and magnitudes and are fully presented in Supplementary File 3.

**Table 2:** Results from an interrupted time series model of proportion of CMD screenings over time.

	*β*	Exp (*β*)	95% CI	*p*-value
Intercept	−1.49	0.22	0.16–0.31	<0.0001
Trend pre-SPE^a^	−0.03	0.97	0.95-0.99	0.004
Immediate change	0.94	2.57	1.34–4.90	0.004
Change in trend post-SPE^b^	0.10	1.11	1.04–1.17	0.001

a 769 visits pre-SPE.

b 385 visits post-SPE.

### 3.3. CMD counselling and referral outcomes

Of 241 visits with a CMD screen, 30 visits (26%) in the pre-SPE period and 36 visits (28%) in the post-SPE period had any counselling at that same visit (*p* = 0.72) (Figure 1). Of the visits that recorded both a screening tool and counselling (*n* = 58), 4 reported elevated depressive symptoms with the PHQ-9 and 4 reported elevated symptoms from the SRQ (Table 3). Counselling topics were predominantly focused on traumatic stress and gender-based violence (pre-SPE: *N* = 27, 3.5% of clinic visits; post-SPE: *N* = 26, 6.8% of clinic visits), with fewer counselling sessions on general topics related to mental health and well-being, such as relationships (pre-SPE: *N* = 2, 0.3%; post-SPE: *N* = 8, 2.1%) and suicidal ideation (pre-SPE: *N* = 1, 0.1%; post-SPE: *N* = 2, 0.5%). Of all visits with a CMD screen, 1 visit (3%) in the pre-SPE period and 6 visits (17%) in the post-SPE period had any counselling at that same visit (*p* = 0.08) (Figure 3). Referrals were made to community health volunteers (pre-SPE, *N* = 1), child psychologists (post-SPE, *N* = 2), and psychiatrists (post-SPE, *N* = 4).

**Table 3:** Counselling and screening tools recorded at visits with a CMD screen (*n* = 241).

	*N* ^a^	% of CMD screens (*n* = 241)^a^
**Counselling recorded** ^b^	**62**	**25.7%**
CAGE-AID/CRAFFT	2	0.8%
PHQ 9	6	2.5%
No depressive symptoms	2	0.8%
Mild	2	0.8%
Moderately severe	1	0.4%
Severe	1	0.4%
PRC	49	20.3%
SRQ	7	2.9%
No depressive symptoms	3	1.2%
Depressive symptoms	4	1.7%
No tool identified^b^	4	1.7%
**No counselling recorded**	**180**	**16.2%**
CAGE-AID/CRAFFT	28	11.6%
PHQ	1	0.4%
No depressive symptoms	1	0.4%
PRC form	1	0.4%
SRQ	9	3.7%
No depressive symptoms	8	3.3%
Depressive symptoms	1	0.4%
No tool identified	140	58.1%

a Sub-categories may not add up to bolded totals if more than one screening tool was used at a clinic visit.

b Four visits reported counselling but no CMD screen at the visit.

## 4. Discussion

In this pilot study of a simulated patient encounter implementation strategy to improve integrated mental health and HIV service delivery in Kenya, we found a significant (*p* < 0.01) association between our SPE training and CMD screenings conducted among AGYW seeking HIV-related services. Specifically, CMD screening proportions more than doubled, from around 8% the week before the SPE training to 21% the week after the SPE training. The proportion of CMD screenings continued to increase over time after the intervention, with an estimated 50% of clinic visits including a CMD screening by the end of the study period suggesting that providers possibly felt more comfortable conducting CMD screens over time. Provider competencies around adolescent-friendly mental health service delivery also improved after training. Our findings highlight the promise of the SPE training approach in promoting CMD service-delivery for adolescents in HIV clinic settings.

Kenyan MOH guidelines recommend screening for depression and alcohol use regularly within HIV care settings (38). While we found a relative increase in the proportion of CMD screens conducted at clinic visits after our SPE-training, a small number of visits overall included screening for depression or anxiety (only 5% of screens after SPE-training) while most of the screening increases seemed to be driven by greater use of substance and alcohol use screening tools. At this clinic, both the substance and alcohol use and the gender-based violence screening tools are integrated into electronic medical records (EMR) while the PHQ-9 and SRQ-20 are done by hand and kept in a patient’s physical file. Additionally, depression and anxiety assessments are mostly done for clients with poor adherence and high viral load. Consequently, we believe that reasons for higher use of substance and alcohol use screening tools include convenience and familiarity of EMR-based tools compared to those for depression and anxiety, concerns about time and effort to deliver screening tools for depression and anxiety, and existing clinic practices for use of depression and anxiety tools. Screening for depression and anxiety, specifically, in HIV care settings is critical to support Kenyan integrated care targets and address the high burden of these conditions among AGYW at risk or living with HIV (39). A study of AGYW in Kenya found that 34% of AGYW with moderate to severe depression had a high HIV risk score (indicating high likelihood of HIV acquisition) (40). One study in Tanzania estimated that 27% of AGYW in an HIV prevention trial had moderate to severe depression, with many reporting experiences of sexual trauma (42%) (41). Despite the evidence of a high burden of depression in this population, no clinic visits screened for depression or anxiety in the 6 months preceding the SPE training and only 5% of clinic visits screened for depression or anxiety after training.

SPEs have been used internationally to improve training in both HIV and mental health care (22). A study using a SPE training for HIV service providers in Kenya found improvements in HIV prevention and PrEP counseling including interpersonal skills, use of guidelines, and adolescent-friendly communication after the training (22, 42). SPE trainings for mental health have also found substantial improvements in CMD screening associated with the training among primary care providers in the United States (43). Our study adds to this body of literature by focusing on care integration and training HIV providers around CMD service delivery specifically.

Several potential mechanisms may explain the improvements seen in the proportion of AGYW clinic visits with a CMD screen after versus before the SPE training. Using an SPE training strategy gives providers space to practice competencies with realistic patients. The SPE training used in our study follows Kolb’s model of experiential learning which includes four stages of learning: (1) concrete experience, or having the actual experience, (2) reflecting on the experience, (3) abstract conceptualization, or learning from the experience, and (4) active experimentation by trying out what was learned (27). We saw marked improvements in provider competencies throughout the training indicating a higher level of provider comfort and familiarity with providing CMD care within HIV care settings. The experiential learning model also supports the continued increases in CMD screening rates we found in the 3 months following training since concrete experience and active experimentation (stages 1 and 4) may lead to increased comfort or knowledge in conducting CMD screenings over time (27).

Approximately one fourth of visits where a CMD screening was performed also included counselling. The most discussed topic of the counselling sessions was traumatic stress and gender-based violence and these visits predominantly reported using the PRC screening tool. AGYW experiencing gender-based violence are at a heightened risk of both HIV and depressive disorders (1, 4, 44). A study of gender-based violence and HIV in Tanzania and South Africa found that 31% of young women screened positive for GBV and only 10% requested referrals (45), highlighting the need for interventions to address these topics within HIV care settings. We found a similar proportion of clinic visits with some counseling conducted (among those when screening for CMDs was also conducted) between the pre- and post-SPE training periods, suggesting that additional support for providers is to address the need for counselling services. Given the high rates of gender-based violence and traumatic stress among AGYW at risk and living with HIV, future studies should look at targeted intervention strategies to address these topics.

While this SPE training model could be expanded to focus on additional topics such as counseling skills and competencies, it is important to note that this study was completed in a context where HIV providers are often limited in their time with a patient. Screening and referrals (both of which occurred more often after the SPE intervention) can be easily integrated into existing HIV care, provided that adequate referral resources exist outside of the clinic. Integrating mental health care (such as counselling) into HIV systems may require further task shifting among HIV care providers, for example training nurse counsellors or staffing an additional cadre of lay providers depending on workload and capacity, to support HIV providers in providing this type of counseling to AGYW (46).

This study had a number of strengths and limitations. Our findings on associations between the SPE training and CMD screening rates were robust and consistent across several sensitivity analyses and sustained through at least 3 months post-intervention. Our SPE training was also rigorously developed based on a formative qualitative process. However, this pilot study included a single clinic and did not include a control group, limiting our ability to detect a causal relationship between CMD screening and SPE training. The time frame of our study prevented us from accounting for seasonality and time-varying confounders such as COVID-19 outbreaks, which may have an impact on a provider’s ability to conduct CMD screening and the need for CMD screening from a patient perspective. Additionally, we cannot evaluate the sustainability of the training after 3 months, or how the effects may be sustained in cases of staff attrition. Our interrupted time series analysis relied on chart abstraction of clinic visits, which may have misclassified information on the outcome of CMD screening tools, details of counselling topics, or other mental health related assessments. For example, clinic visits were categorized as having no CMD screen if none was found in the abstracted medical record, but it is possible that providers did perform a screen and did not record it. It is also possible that providers conducted CMD screenings and counselling before the SPE intervention but recorded them with more accuracy post-training. To the extent that was the case, our findings would be biased to show a larger effect of the SPE training than truly occurred. Although we did not measure accuracy of recorded medical records, improved reporting of CMD screenings in medical records could be considered an unintended benefit of this intervention as it may help with resource allocation, follow-up, or appropriate referral. Finally, our analysis only looked at changes in the proportion of CMD screens (and any counseling or referrals provided). We did not collect data on quality of mental health care, fidelity to the mental health training post-SPE, or clinical outcomes such as receipt of mental health psychotherapy or pharmacotherapy, improved CMD symptoms, or improved adherence to HIV prevention or treatment care in our pilot study. It is also important to understand if actions taken after screening were consistent with screening outcomes. Therefore, future work is needed to consider clinical and implementation outcomes related to the provision of adequate, integrated mental health and HIV services for AGYW (1).

In conclusion, our SPE model is a promising implementation strategy for improving HIV provider competencies and CMD service delivery for adolescents in HIV clinics. Gaps remain, however, around high burden CMDs, such as depression, and achieving MOH targets of screening all AGYW for CMDS at HIV service delivery appointments. Additional research is needed to explore long-term effects of SPE training on adolescent clinical outcomes in larger trials. Future SPE training approaches could incorporate targeted training on high burden conditions (e.g., depression, anxiety) among AGYW seeking HIV services.

## Data availability statement

The raw data supporting the conclusions of this article will be made available by the authors, without undue reservation.

## Ethics statement

The studies involving humans were approved by University of Washington and Kenya Medical Research Institute Scientific and Ethics Review Unit. The studies were conducted in accordance with the local legislation and institutional requirements. The ethics committee/institutional review board waived the requirement of written informed consent for participation from the participants or the participants’ legal guardians/next of kin because Kenyan age of consent is 16 years and therefore parental consent was not needed for participants between ages 16 and 18. No participants were included under the age of 16.

## Author contributions

JV and KN conceptualized the research and methodology and acquired funding. RN, BK, EO, and GM were responsible for data collection and management. PM, KN, NM, and CK conducted project oversight and operational management. PK, SD, and PC advised on the actor case scripts and the SPE training intervention. TC and JV conducted the analysis and wrote the original draft of the manuscript. BW contributed to analysis approach and interpretation. All authors contributed to the article and approved the submitted version.

## Funding

This research was funded by a pilot award from the University of Washington Global Mental Health Program. JV was supported by the National Institute of Mental Health (NIMH; Grant K99 MH123369 and Grant R00 MH123369). Data collection through RedCap was supported by the Institute of Translational Health Science (ITHS) grant support (UL1 TR002319, KL2 TR002317, and TL1 TR002318 from NCATS/NIH).

## Conflict of interest

The authors declare that the research was conducted in the absence of any commercial or financial relationships that could be construed as a potential conflict of interest.

## Publisher’s note

All claims expressed in this article are solely those of the authors and do not necessarily represent those of their affiliated organizations, or those of the publisher, the editors and the reviewers. Any product that may be evaluated in this article, or claim that may be made by its manufacturer, is not guaranteed or endorsed by the publisher.
