# Supplementary material for: Higher rates of mental health screening of adolescents recorded after provider training using simulated patients in a Kenyan HIV clinic: results of a pilot study
Source: Front Public Health. 2023 Sep 22;11:1209525. doi: 10.3389/fpubh.2023.1209525 (PMC10556463; doi:10.3389/fpubh.2023.1209525)
Supplement: Supplementary file 1 [file Data_Sheet_1.docx]

**Standardized Patient Actor Script: Case 1**

**Case 1:** AGYW seeking HIV treatment

**Visit:** ART refill and viral load assessment

| **BACKGROUND CHARACTERISTICS:** | |
| --- | --- |
| **Service(s) seeking:** | HIV treatment (ART services) |
| **Patient name:** | Sally Akinyi |
| **Patient demographics:**  *Age*  *Date of birth*  *Sex*  *Height*  *Weight* | 22 years  01 Jan 1999  Female  [Actual]  [Actual] |
| **HIV status:** | HIV-positive |
| **Materials/equipment needed:** | screening tool SRQ/PHQ writing material |

| **CASE GOAL:** |
| --- |

The goal of this case is to assess the knowledge and skills of an HIV provider related to conducting screening for common mental disorders, identifying whether a referral for further mental healthcare is needed, and providing basic counseling for a young women seeking HIV treatment and viral load measurement.

| **COMPETENCIES TESTED:** |
| --- |
| Provider should be able to: provide ART and adherence counseling; screen for common mental disorders; assess next steps in care based on screening results; conduct basic counseling using problem-solving skills (as needed) including helping the client identify and prioritize problems and develop an action plan; make an appropriate referral for further mental health care as needed; exhibit active listening skills; and ask clarifying questions. |

| **CASE SUMMARY:** | | | |
| --- | --- | --- | --- |
| **Age:** | | 22 years | |
|  | **Visit:**  *Seeking an ART refill, it has been 6 months since her HIV diagnosis* | |  |
| **Visit purpose:** | | - Seeking ART refll | |
| **Main concerns:** | | - Coming to terms with recent HIV diagnosis - Inadvertent disclosure of HIV status - Fear of disclosure - Persistent feelings of being distressed. | |
| **Partner(s):** | | One current sexual partner | |
| **Risk factors:** | | - Has had difficulties complying to treatment - Fears reaction from family and friends and has not received support from family | |
| **Sexual behaviors:** | | One current sexual partner but also has had a casual partner in the past 6 months | |
| **Health factors:** | | - HIV-positive - High viral load - Depressive symptoms - Struggling to take ART well | |
| **Children:** | | None | |

**Your challenge as a standardized patient (SP) actor is to:**

1. Appropriately and accurately respond to questions related to your case.
2. Accurately recall the encounter during the briefing session

| **SP OPENING STATEMENT:** |
| --- |
| When you arrive at the clinic, you will approach the health care provider in his / her clinic room. He or she will ask you why you have come for clinic and you will respond as stated below. You must say the opening statement verbatim. Do not paraphrase.  When asked by the health care provider “why have you come to the clinic today” or “how can I help you?” your response should be:  “*Doctor, I am here today to pick up my HIV medication.*”  As the health care provider carries out their routine follow up visit assessment, wait to see if they ask you about your mental well-being.  **[If asked about your mental well-being:]**  If the health care provider asks you if you have any issues in regards to your mental well-being state:  ***“****I still can’t* *understand where I could have contacted the virus since I have never been a person who is loose. I don’t think this result is right and I’m having trouble taking my medication; my family will harm and disown me if they find out about this. I cry all the time, and I’m hopeless.”*  **[If NOT asked about your mental well-being:]**  If the healthcare provider does not ask you about your mental well-being, then ask:  ***“****Do you advice people here?’’*  Wait for them to tell you about it, then respond:  ***“****I feel bad and I need to talk to someone who will understand me.****”***  **[If screened for mental health concerns:]**  If you are screened for mental health concerns and depressive symptoms, please endorse items related to the following symptoms:   - *Feeling down, depressed, or hopeless* - *Crying a lot* - *Self-blame, feeling bad about yourself* - *Headaches* - *Difficulty sleeping or change in sleep patterns* - *Difficulty eating or change in appetite* - *Alcohol use* |

| **PRESENTATION & EMOTIONAL TONE:** | |
| --- | --- |
| Present in a tired state. You are walking and sitting normally but you keep your eyes down and are reluctant to engage with the provider. You sit with a slouched posture with your arms folded and look distressed, anxious, and shy.  Dress in a manner that would be considered appropriate and similar to others in the area of your age and gender, such as a t-shirt with jeans or medium length skirt.  You are a bit disheveled state and look as if you have been trouble sleeping lately. | |
| **Responding to questions:** | You are hesitant and slow to respond to questions. You have a change of facial expression anytime HIV is mentioned and appear particularly disturbed when discussing matters of disclosure of HIV. When the healthcare provider asks questions about HIV acquisition or mentions a high viral load, you break down. You are devastated by your report of a high viral load. |
| **Changes in demeanor during the encounter:** | During the encounter when presented with questions about HIV, answer in a monotone fashion and only respond when prompted. Cry during the session and start to respond positively when the provider makes supportive statements. Nod in agreement and acknowledgement as the provider states symptoms of depression. |
| By the end of the encounter, if the provider asks thoughtful and supportive questions (for example, a thoughtful or supportive statement could be, “I know it can be hard for young women to talk about these issues – you’re doing great. Can you tell me a bit more about what you are feeling?”), you start to warm-up and look more interested interested and engaged. | |

| **HISTORY OF REASONS TO SEEK ART:** |
| --- |
| You had been dating your main partner for a year. Overall, you believe he is a good boyfriend. You are not sure if your boyfriend has other partners, but you suspect he does because he often goes away and does not explain where he has been. You had asked him about his HIV status but he has never been tested and does not know his status. You asked him to do a couples test with you but he refused. When you have brought this up in the past, he feels you are accusing him of being unfaithful and cuts the conversation short. You also have another partner who you have been casually seeing in the past year (you usually see him when your boyfriend is out of town or when you are fighting with your boyfriend). You do not know your casual partner’s HIV status either. You always use condoms with your casual partner but rarely use condoms with your main boyfriend.  You went to the doctor for contraceptive services six months ago and did an HIV test while you were there. You found out that you were HIV positive and had a high viral load. Since then you have not been back to the clinic for ART refills or viral load testing because you are having trouble accepting your HIV status. You decided that you would like more help managing your HIV infection so you have come back to the clinic today.  **Your current concern is:**  You are struggling with your HIV diagnosis: You recently tested positive for HIV but you are struggling to accept the results. You cannot imagine how your family members will react and fears to disclose the results citing your safety and not being accepted.  You report feeling hopeless, and having poor sleep, poor appetite and low moods. You score highly on a depressive symptom screening tool, reporting these symptoms as well as alcohol use. |

| **PAST MEDICAL HISTORY:** | |
| --- | --- |
| **Overall health:** | Lost 10% of body weight over the past six weeks and currently has poor sleep, appetite and low moods. Seeking ART refills and support from the CCC. |
| **Prior illness:** | No significant past medical issues. |
| **Prior ART use:** | Prescribed ART 6 months ago but has had trouble adhering |
| **STI screening:** | None |
| **Allergies:** | None |
| **Past hospitalizations:** | No past history of admission. |

| **SEXUAL HISTORY**  Has had one casual partner over the past year but currently in a stable relationship with current partner. |
| --- |

| **MEDICATIONS:** | |
| --- | --- |
| **Prescription drugs:** | None |
| **Family planning:** | Hormonal IUD |
| **Over the counter (OTC) drugs, herbal or traditional medicines:** | Uses piriton because of poor sleep |
| **Illicit/street drugs:** | None |

| **PERSONAL HISTORY:** | |
| --- | --- |
| **Birth date:** | 01 Jan 1999 |
| **Birth place:** | Nairobi |
| **Religion:** | Christian |
| **Alcohol:** | Thrice a week |
| **Tobacco:** | None |
| **Caffeine:** | Takes tea once per day |
| **Diet:** | You eat a normal diet for the area (ugali, rice, githeri, vegetables, fruits). |
| **Exercise:** | None |
| **Activities/hobbies/social life:** | No social life but attends Chama meetings |
| **Stress:** | People knowing you are HIV positive  Cannot imagine how she will live with HIV  Failing health, high viral load  Feels distressed, poor sleep, crying all the time, poor appetite, low energy level |
| **Occupation:** | Bar waiter |
| **Education:** | Form 4. |
| **Living arrangement:** | Lives alone in a small house |

| **FAMILY HISTORY:** | |
| --- | --- |
| **Parents:** | Single parent who died of cancer. |
| **Siblings:** | Has 6 siblings; there is history of depressive illness in two of the siblings |
| **Children:** | None |
| **Parental grandparents:** | Grandmother Alive ,Grandfather Dead |
| **Maternal grandparents:** | None |

| **AT THE END OF THE ENCOUNTER:** |
| --- |
| After the healthcare worker has completed the examination,   - Standardized checklists will be used to evaluate the healthcare provider encounter with the SP actor. - The SP actor will complete checklists based on individual scenarios to provide feedback for the healthcare provider on their practice session and provide a score based on competencies met. |

**Standardized Patient Actor Scripts: Case 2**

**Case** Young woman, seeking pre-exposure prophylaxis

**Visit 1:** PrEP initiation

| **BACKGROUND CHARACTERISTICS:** | |
| --- | --- |
| **Service(s) seeking:** | Pre-exposure prophylaxis |
| **Patient name:** | Mary Nyaboke |
| **Patient demographics:**  *Age*  *Date of birth*  *Sex*  *Height*  *Weight* | 24 years  01 Mar 1997  Female  [actual]  [actual] |
| **HIV status:** | Unknown |
| **Materials/equipment needed:** | None |

| **CASE GOAL:** |
| --- |
| The goal of this case is to assess the knowledge and skills of an HIV provider related to conducting screening for common mental disorders, identifying whether a referral for further mental healthcare is needed, and providing basic counseling for a young women seeking pre-exposure prophylaxis. |

| **COMPETENCIES TESTED:** |
| --- |
| Provider should be able to: provide basic information about PEP; assess for behavioral HIV risk; provide counseling on PEP side effects and HIV; screen for common mental disorders; assess next steps in care based on screening results; conduct basic counseling using problem-solving skills (as needed) including helping the client identify and prioritize problems and develop an action plan; make an appropriate referral for further mental health care as needed; exhibit active listening skills; and ask clarifying questions. |

| **CASE SUMMARY:** | | |
| --- | --- | --- |
| **Age:** | 24 years | |
|  | **Visit 1:**  *Seeking PrEP for the first time* | |
| **Visit purpose:** | | - Seeking Pre -exposure prophylaxis (PrEP) - Client is concerned about risks associated with partner’s sexual behaviour. |
| **Presenting issues:** | | - Fighting with partner concerning cheating on her - Partner and parents financially dependent on her - One child living in the rural area with her parents - Suicidal thoughts |
| **Partner(s):** | | One |
| **Risk factors for HIV transmission or acquisition:** | | - Partner cheating on her (concerned about sexual behavior of partner) |
| **Risk factors for common mental disorder:** | | - Emotionally unstable due to fights with partner - Engaging in fights with partner due to unfaithfulness in the relationship - Financial constraints - Having suicidal thoughts due to all the above |
| **Sexual behaviors:** | | In a relationship where partner is unfaithful |
| **Health factors:** | | - No symptoms or history of STIs - HIV-negative at her last test 4 months ago - Looking depressed - Emotionally not stable - No history of alcohol |
| **Children:** | | Has a child living with parents at the rural home |

**Your challenge as a standardized patient (SP) actor is to:**

1. Appropriately and accurately respond to questions related to your case.
2. Accurately recall the encounter during the briefing session.

**Visit 1: Seeking PrEP**

| **PATIENT OPENING STATEMENT:** |
| --- |
| When you visit health facility, the first contact person will be front office manager. Due to COVID rules in place you will be triaged before meeting a health provider. When the clinician is available, you will be sent into the clinician’s room. The clinician will ask you why you came to the clinic and you will respond as stated below.  You must say the opening statement verbatim. Do not paraphrase.  When asked by the health provider care provider, “Why have you come to the clinic today?” or “How can I help you?”, your response should be:  **“*I am here today because I am concerned about getting HIV from my partner -- my partner is cheat.”*unkem toe the clinic to ardIV. I have heard of this PrEP medication a friend talked about. unkem toe the clinic to ardIV. I have heard of this PrEP medication a friend talked about.**  unkem toe the clinic to ardIV. I have heard of this PrEP medication a friend talked about.  As the health care provider carries out their routine follow up visit assessment, wait to see if they ask you about your mental well-being.  **[If asked about your mental well-being:]**  If the health care provider asks you if you have any issues in regards to your mental well-being, reluctantly state:   - *“Yes, I have issues.”*   If the provider asks for more information respond:   - *“I am feeling very stressed.”*   Only provide additional details about the sources of stress (e.g., “*I am the breadwinner for my family”; “I am taking care of my parents at home”; “I have a son who is staying with my parents”; or “my partner is not supportive financially”)* if specifically asked by the provider.  **[If NOT asked about your mental well-being:]**  If the healthcare provider does not ask you about your mental well-being, then ask:  *“I would like to talk to a counsellor. Is there a counselor available whom I can share with my personal issues?*  Wait for them to tell you about it, then respond:  *“ I feel very stressed and I need to talk to a counsellor who can advise me”*  **[If screened for mental health concerns:]**  If you are screened for mental health concerns and depressive symptoms, please endorse items related to the following symptoms:   - *Feeling down, depressed, or hopeless* - *Crying a lot* - *Self-blame, feeling bad about yourself* - *Headaches* - *Difficulty sleeping or change in sleep patterns* - *Suicidal ideation and intent* |

| **PRESENTATION & EMOTIONAL TONE:** |
| --- |

| Walk in a provider’s room feeling down because you are unable to express your feelings. Stand before the provider speechless waiting for their support.  Dress in a manner that would be considered appropriate and similar to others in the area of your age and gender, such as a t-shirt with jeans or medium length skirt.  You appear a bit disheveled and your eyes are puffy and red. You also appear confused and hesitant. | |
| --- | --- |
| **Responding to questions:** | Respond to provider’s questions with closed ended answers. When provider asks about sexual history, HIV risk and mental health act emotionally unstable (cracking your knuckles, shaking head, frowning/worried expression on your face, tone of voice breaks as if you are about to cry). |
| **Changes in demeanor during the encounter:** | When provider address sexual history and HIV concerns node your head and answer in low tone; show willingness to respond. Give verbal consent to give personal information and respond to HIV and sexual history questions as you seek clarification of what has been asked. |
| Appreciate the provider for their concern and care as you feel you have been heard and supported by the provider. Discuss freely on issues to do with why you considered suicidal thoughts, your HIV risk and mental health. Then give appreciation for PrEP dispensed and any other services accorded. | |

| **HISTORY OF REASONS TO SEEK PrEP AND MENTAL HEALTH SUPPORT** |
| --- |
| You are in a relationship and your partner is cheating on you. You have a feeling that you are at risk of HIV. You learned of PrEP medication from a friend. You are at the clinic to be dispensed. You are a casual labourer with no consistent source of income. On a good day you earn KES 200 per day and that money is supposed to sustain you and your partner as he doesn’t have a job and relies on you financially. In addition, your parents who live in the rural area expect you to send them money every month to support them since they are old and also because they are taking care of your son. You just feel overwhelmed with your current circumstances and that’s part of reasons you describe feelings of suicidal intent and ideation.  **Your current concern is:**  Fear of contracting HIV since your partner is exposing you by cheating, and financial pressure you feel from your partner and your parents. The problems you are facing are related to feelings of suicidal ideation which worry you. |

| **PAST MEDICAL HISTORY:** | |
| --- | --- |
| **Overall health:** | Looking unkept due to harsh economic situation. |
| **Prior illness:** | No history of recurring illness |
| **Prior PrEP/PEP/ART use:** | None |
| **HIV/AIDS screening:** | You last HIV tested about six months ago and are here for a repeat HIV test so you can pick up PrEP medication |
| **Common mental disorder screening:** | You have never been screened for common mental disorders or sought mental health care. However, you are concerned about your feelings of suicidal ideation and want to seek care now. |
| **GBV screening:** | You have never experienced sexual assault or gender-based violence. |
| **STI screening:** | You have never been treated for any STIs and have never experienced any symptoms. You do not know if any of your partners have ever had any STIs. |
| **Allergies:** | None |
| **Past hospitalizations:** | Never been hospitalized or had any surgeries |

| **SEXUAL HISTORY:** |
| --- |
| You had a boyfriend who is the father to your child and separated 3 years back. Currently in a relationship with a man aged 28 years he drinks heavily and has many sexual partners. Engages in casual sex when drunk. |

| **PERSONAL HISTORY:** | |
| --- | --- |
| **Birth date:** | 01 Mar 1997 |
| **Birth place:** | Kisii County, Keroka sub- sub county |
| **Religion:** | Christian |
| **Alcohol:** | Doesn’t drink |
| **Tobacco:** | Do not use |
| **Caffeine:** | You drink a cup of tea 1-3 times per day |
| **Diet:** | You eat a normal diet for the setting (Ugali, matoke, rice and greens) |
| **Exercise:** | You do not exercise |
| **Activities/hobbies/social life:** | You enjoy supporting the needy, traveling, and meeting new friends. |
| **Stresses:** | You are worried about HIV infection. Not happy with current financial state and being emotionally unstable (especially suicidal feelings). |
| **Occupation:** | Casual labourer |
| **Education:** | Completed high school. |
| **Living arrangement:** | Living with partner |
| **Exposure to violence:** | You have a history of violence by a partner. You fear of what the future will be like. |

| **FAMILY HISTORY:** | |
| --- | --- |
| **Parents:** | Your mother is healthy with no medical problems as far as you know. Dad has history of urine incontinence. |
| **Siblings:** | You are from a family of 5 siblings. |
| **Children:** | You are a mother of one child |
| **Parental grandparents:** | Not alive, they died before you were born |
| **Maternal grandparents:** | Died of post-election violence. |

| **MEDICATIONS:** | | |
| --- | --- | --- |
| **Prescription drugs:** | | You are currently not on any medications. |
| **Family planning:** | | IUD |
| **Over the counter (OTC) drugs, herbal or traditional medicines:** | | None |
| **Illicit/street drugs:** | | None |
| **MENSTRUAL HISTORY:** | | |
| Premenopausal |  | |

| **AT THE END OF THE VISIT:** |
| --- |
| After the healthcare worker has completed the examination, you should do the following:   1. Standardized checklists will be used to evaluate the healthcare provider encounter with the SP actor. 2. The SP actor will complete checklists based on individual scenarios to provide feedback for the healthcare provider on their practice session and provide a score based on competencies met. |

**Standardized Patient Actor Scripts: Case 3**

**Case 3:** Young woman, seeking post-exposure prophylaxis

**Visit 1:** PEP initiation

| **BACKGROUND CHARACTERISTICS:** | |
| --- | --- |
| **Service(s) seeking:** | Post-exposure prophylaxis |
| **Patient name:** | Mercy Wangeci |
| **Patient demographics:**  *Age*  *Date of birth*  *Sex*  *Height*  *Weight* | 18 years  27 Feb 2003  Female  [actual]  [actual] |
| **HIV status:** | Unknown |
| **Materials/equipment needed:** | None |

| **CASE GOAL:** |
| --- |
| The goal of this case is to assess the knowledge and skills of an HIV provider related to conducting screening for common mental disorders, identifying whether a referral for further mental healthcare is needed, and providing basic counseling for a young women seeking post-exposure prophylaxis after a sexual assault. |

| **COMPETENCIES TESTED:** |
| --- |
| Provider should be able to: provide basic information about PEP; assess for behavioral HIV risk; provide counseling on PEP side effects and HIV; screen for common mental disorders; assess next steps in care based on screening results; conduct basic counseling using problem-solving skills (as needed) including helping the client identify and prioritize problems and develop an action plan; make an appropriate referral for further mental health care as needed; exhibit active listening skills; and ask clarifying questions. |

| **CASE SUMMARY:** | | |
| --- | --- | --- |
| **Age:** | 18 years | |
|  | **Visit 1:**  *Seeking PEP for the first time* | |
| **Visit purpose:** | | - Seeking post-exposure prophylaxis (PEP) - Concerned she may have acquired HIV |
| **Main concerns:** | | - You have recently experienced sexual assault - You are concerned you may have acquired HIV but are hesitant to discuss the details of the experience with a provider for fear of stigma and provider reaction |
| **Partner(s):** | | None |
| **Risk factors for HIV transmission or acquisition:** | | - Experienced sexual assault, and HIV status of perpetrator(s) is unknown |
| **Risk factors for common mental disorder:** | | - Experienced sexual assault, but otherwise no pre-existing conditions |
| **Sexual behaviors:** | | Previously monogamous with a partner (they broke up a year ago) |
| **Health factors:** | | - No symptoms or history of STIs - HIV-negative at her last test (one year ago) - Not currently pregnant or trying to become pregnant - Occasionally drinks alcohol (frequency has increased recently since the sexual assault) - Occasionally smokes bhang (frequency has increased recently since the sexual assault) |
| **Children:** | | None |

**Your challenge as a standardized patient (SP) actor is to:**

1. Appropriately and accurately respond to questions related to your case.
2. Accurately recall the encounter during the briefing session.

**Visit 1: Seeking PEP**

| **PATIENT OPENING STATEMENT:** |
| --- |
| When you arrive at the clinic, you will first approach the provider at the reception desk. He or she will ask you why you came to the clinic then send you to the clinician’s room. The clinician provider will ask you why you came to the clinic and you will respond as stated below.  You must say the opening statement verbatim. Do not paraphrase.  When asked by the provider “why are you at the clinic today” or “how can we help you”, your response should be:  ***“I was wondering if you provide people with PEP here.”***  As the provider assists you with PEP, wait and see if they ask you about your interest in initiating PEP for HIV prevention, your reasons for feeling at risk for HIV and your current feelings.  **[If asked about PEP:]**  If the provider asks you if you have heard of PEP before and your reasons for asking for it today, state:   - *“Yes, I have heard of PEP in the radio, could you explain to me more?”* - *“I would like to use it to prevent myself from HIV infection due to a rape incidence.”*   If the provider asks you how you are feeling today or about any mental health concerns, state:   - *“I feel scared that I may have been infected by HIV. I feel very sad that someone I thought I knew did this to me.”*   **[If NOT asked about PEP:]**  If the provider does not ask you about your interest in PEP, your reasons for asking for it and your feelings today, then ask:  ***“I once heard in the radio that someone should take PEP immediately after a rape incidence to prevent HIV infection, could you tell me more about it?”***  Wait for them to tell you about it then respond:   - ***“Would PEP prevent me from being infected by HIV?*** - ***“I feel scared that I may have been infected by HIV. I feel very sad that someone I thought I knew did this to me.”***   **[If screened for mental health concerns:]**  If you are screened for mental health concerns and depressive symptoms, please endorse items related to the following symptoms:   - *Feeling down, depressed, or hopeless* - *Crying a lot* - *Self-blame, feeling bad about yourself* - *Headaches* - *Difficulty sleeping or change in sleep patterns* - *Difficulty eating or change in appetite* - *Alcohol use* - *Substance use* |

| **PRESENTATION & EMOTIONAL TONE:** |
| --- |

| Present in a shy and nervous state. You are walking and sitting normally but you keep your eyes down and are reluctant to engage with the provider. You are anxious about HIV testing and to talk about the sexual assault experience because you are feeling depressed, hopeless, and sad about it.  Dress in a manner that would be considered appropriate and similar to others in the area of your age and gender, such as a t-shirt with jeans or medium length skirt.  You are well put-together, but look like a student. | |
| --- | --- |
| **Responding to questions:** | You are responsive and answer all of the healthcare provider questions when asked according to your case. You do not share information spontaneously. When the healthcare provider asks questions regarding sex, HIV, or mental health you should act uncomfortable with the topic, but not extremely so. |
| **Changes in demeanor during the encounter:** | During the middle of the encounter when presented with questions regarding sex, HIV, or mental health respond in a hesitant manner, somewhat anxious with the topic but willing to respond. If discussed, you agree it is a good idea to answer questions about your mental health and wellbeing and to talk with a counselor about it. |
| At the end of the encounter, you should act grateful for the PEP delivery, opportunity to talk about your mental health symptoms, and referral and other information about depression and alcohol/substance use issues. | |

| **HISTORY OF REASONS TO SEEK PrEP/PEP/ART OR MENTAL HEALTH SERVICES:** |
| --- |
| You have been out of a relationship after a bad breakup with your boyfriend one year ago. You caught him cheating on you with your best friend. You went to the nearby VCT (Voluntary counselling and Testing Centre) and got an HIV test that was negative. You decided to keep off relationships until someday. Last week your brother’s friend invited you to an all-night house party. Since you enjoy parties, you accepted the invitation. When you arrived, there were many young people like you there, some whom were familiar. Your brother was not around as he had travelled to Qatar for work last month. You were excited to have fun and meet new people and maybe find somebody that you like there to be your new boyfriend.  As the party progressed, you took more and more alcohol. Your brother’s friend was extremely attentive to you and he kept refilling your glass. You danced a lot but at some point you felt drowsy and asked your brother’s friend to show you a place to lie down. He carried you to his spare bedroom and later you woke up startled by somebody’s hands pulling off your clothes. The house was quiet and it seemed everybody had left. You looked up only to see your brother’s friend forcing himself on you. You tried to scream but he silenced you with a heavy slap to your face. He covered your mouth with a cloth and shouted at you to be quiet or face worse consequences. You are not sure if he used condoms. When he was you scrambled to put on your clothes and ran away from the place shaken. A cab driver took you home.  **Your current concern is:**  You are concerned that you may have acquired HIV: Your brother’s friend raped you and you are not sure if he used condoms. You suspect your brother’s friend could be HIV-positive as you are aware that he has many partners. |

| **PAST MEDICAL HISTORY:** | |
| --- | --- |
| **Overall health:** | Your last visit to a health facility was about a year ago for a stomach ache and an HIV test. You are otherwise healthy and do not have symptoms related to acute HIV. You had all of the normal childhood diseases and vaccinations. You have no known drug allergies. |
| **Prior illness:** | No prior illnesses |
| **Prior PrEP/PEP/ART use:** | None |
| **HIV/AIDS screening:** | You last tested for HIV one year ago, during a time when you had been in a monogamous relationship. You have not received your HIV test results yet, at the time of this patient encounter. |
| **Common mental disorder screening:** | You have never been screened for common mental disorders or sought mental health care. However, you have previously felt similar feelings of being down and hopeless, when your father died about 5 years ago. During that time, you also drank a lot and went out with friends to try and make yourself feel better. Since your assault, you have been unable to sleep or eat well. You have frequent headaches for which you take painkillers. You also cry a lot and blame yourself. You smoke ‘bhang’ to try and forget the assault memory. |
| **GBV screening:** | You have never experienced sexual assault or gender-based violence before this instance. |
| **STI screening:** | You have never been treated for any STIs and have never experienced any symptoms. You do not know if any of your partners have ever had any STIs. |
| **Allergies:** | None |
| **Past hospitalizations:** | Never been hospitalized or had any surgeries |

| **SEXUAL HISTORY:** |
| --- |
| You dated your previous boyfriend for about two years. He had been your first and only sexual partner. During the relationship, he refused to go for HIV testing with you, accusing you of not trusting him. You did not want to lose him so you abandoned the idea. Later you found him cheating on you with your best friend and you broke up with him. You went for a HIV test soon after and found out that you were HIV-negative. You did not go for a follow up HIV test after 3 months as advised by the HIV provider.  You have not had a serious boyfriend since then but had casual sex with two other young men not long after you broke up with your boyfriend.  You do not tell the health care provider about your past sexual partners unless you are specifically asked how many sex partners you have had (total = 3). |

| **PERSONAL HISTORY:** | |
| --- | --- |
| **Birth date:** | 27 Feb 2003 |
| **Birth place:** | Murang’a County, Kangema sub-county |
| **Religion:** | Christian |
| **Alcohol:** | You drink alcohol occasionally. |
| **Tobacco:** | You do not smoke tobacco but smoke ‘bhang’ occasionally. |
| **Caffeine:** | You drink a cup of tea 1-2 times per day. |
| **Diet:** | You eat a normal diet for the area (Rice, beans, ‘githeri’, potatoes, meat, fruits and vegetables). |
| **Exercise:** | You do not exercise. |
| **Activities/ hobbies/ social life:** | You enjoy watching movies, spending time with your family, visiting friends and going for parties. You have a small group of friends but like meeting new people. |
| **Stresses:** | You are worried about HIV infection. You are also worried about what people will think about you if they find out about your sexual assault. You are afraid of being hurt again by your brother’s friend and are afraid of leaving your house. |
| **Occupation:** | Unemployed. |
| **Education:** | Completed high school. |
| **Living arrangement:** | Live with your mother and two younger sisters. |
| **Exposure to violence:** | No history of violence by a partner or other person before this instance. You fear that your brother’s friend or his friends could hurt you again if they heard that you reported them to the authorities. |
| **FAMILY HISTORY:** | |
| **Parents:** | Your mother is healthy with no medical problems as far as you know. Your father died about five years ago in a road traffic accident. |
| **Siblings:** | You have one older brother and two younger sisters. They are healthy with no medical problems as far as you know. |
| **Children:** | None. You would like to have children in the future, but would like to be married first. |
| **Parental grandparents:** | Deceased; Grandmother died of a lower respiratory infection. Grandfather died of unknown cause but was old. |
| **Maternal grandparents:** | Deceased; Grandmother died of cancer. Grandfather died of tuberculosis. |

| **MEDICATIONS:** | | |
| --- | --- | --- |
| **Prescription drugs:** | | You are currently not on any medications. |
| **Family planning:** | | Injectable contraception |
| **Over the counter (OTC) drugs, herbal or traditional medicines:** | | None. |
| **Illicit/street drugs:** | | Bhang |
| **MENSTRUAL HISTORY:** | | |
| Premenopausal |  | |
| **AT THE END OF THE VISIT:** | | |
| After the healthcare worker has completed the examination:   1. Standardized checklists will be used to evaluate the healthcare provider encounter with the SP actor. 2. The SP actor will complete checklists based on individual scenarios to provide feedback for the healthcare provider on their practice session and provide a score based on competencies met. | | |

**Standardized Patient Actor Script: Case 4**

**Case 4:** Young woman, seeking antiretrovirals for HIV treatment

**Visit 1:** ART routine follow up visit

| **BACKGROUND CHARACTERISTICS:** | |
| --- | --- |
| **Service(s) seeking:** | HIV treatment (ART services) |
| **Patient name:** | Natalie Nasenya |
| **Patient demographics:**  *Age*  *Date of birth*  *Sex*  *Height*  *Weight* | 16 years  4 Sep 2004  Female  [Actual]  [Actual] |
| **HIV status:** | HIV-positive |
| **Materials/equipment needed:** | None |

| **CASE GOAL:** |
| --- |

The goal of this case is to assess the knowledge and skills of an HIV provider related to conducting screening for common mental disorders, identifying whether a referral for further mental healthcare is needed, and providing basic counseling for a young women seeking HIV treatment.

| **COMPETENCIES TESTED:** |
| --- |
| Provider should be able to: provide basic information about ART; screen for common mental disorders; assess next steps in care based on screening results; conduct basic counseling using problem-solving skills (as needed) including helping the client identify and prioritize problems and develop an action plan; make an appropriate referral for further mental health care as needed; exhibit active listening skills; and ask clarifying questions.  . |

| **CASE SUMMARY:** | |
| --- | --- |
| **Age:** | 16 years |
| **Visit purpose:** | ART follow up visit. |
| **Main concerns:** | You are currently pregnant and are struggling to accept the pregnancy.  Your father has refused to pay your school fees resulting to you dropping out. The father claims that you are not his daughter.  You have been having fights with your parents and your serious boyfriend due to the unplanned pregnancy.  Disclosure of your HIV status to your serious boyfriend. |
| **Partner(s):** | You have a serious boyfriend of unknown HIV status. |
| **Risk factors:** | - Poor adherence to ART - Fear of having infected the boyfriend with HIV - Feels abandoned and alone |
| **Sexual behaviors:** | One partner |
| **Health factors:** | - HIV-positive - Detectable viral load of about 1500 copies - No history of AIDS - No history of diabetes |
| **Children:** | None |

**Your challenge as a standardized patient (SP) actor is to:**

1. Appropriately and accurately respond to questions related to your case.
2. Accurately recall the encounter during the briefing session.

| **PATIENT OPENING STATEMENT:** |
| --- |
| When you arrive at the clinic, you will approach the health care provider in his / her clinic room. The clinician provider will ask you why you came to the clinic and you will respond as stated below.  You must say the opening statement verbatim. Do not paraphrase.  When asked by the health care provider “why have you come to the clinic today” or “how can I help you?”, your response should *be:*  ***“I have come for my routine follow up visit.”***  As the health care provider carries out their routine follow up visit assessment, wait to see if they ask you about your mental well-being.  **[If asked about your mental well-being:]**  If the health care provider asks you if you have any issues in regards to your mental well-being state:   - ***“I don’t know where to start but I feel like everybody has left me and nobody wants me. I am lost and alone”***   **[If NOT asked about your mental well-being:]**  If the healthcare provider does not ask you about your mental well-being, then ask:  ***“Do you offer any counselling or can I talk to someone?”***  Wait for them to tell you about it, then respond:  ***“I am interested as I am currently facing a lot of problems in my life”***  **[If screened for mental health concerns:]**  If you are screened for mental health concerns and depressive symptoms, please endorse items related to the following symptoms:   - *Feeling down, depressed, or hopeless* - *Crying a lot* - *Feeling lonely and isolated* - *Self-blame, feeling bad about yourself* |

| **PRESENTATION & EMOTIONAL TONE:** | |
| --- | --- |
| Present in a somewhat restless and gloomy state. You are walking and sitting normally but you look restless and deep in thoughts. You are uncomfortable as you have missed your pills for some days due to whatever is currently happening in your life.  Dress in a manner that would be considered appropriate and similar to others in the area of your age and gender, such as a colorful top and a skirt. | |
| **Responding to questions:** | You are responsive and answer all of the healthcare provider questions when asked according to your case. You do not share information spontaneously. When the healthcare provider asks questions regarding sex and mental health you should act quite uncomfortable with the topic. |
| **Changes in demeanor during the encounter:** | During the encounter when presented with questions regarding sex, mental health or drug use, respond in a hesitant manner, uncomfortable with the topic but willing to respond. If discussed, you agree it is a good idea to talk more regarding these issues as long as it remains confidential. |
| At the end of the encounter, you should act grateful for the ART services provided and information about your mental health and any referrals. | |

**Your current concern is:**

| You are currently pregnant and your father has refused to pay school fees resulting to you dropping out of school. You have been having fights with your parents and your boyfriend.  You are unsure how to disclose your status to your partner and have had difficulty accepting your pregnancy status. |
| --- |

| **HISTORY OF REASONS TO SEEK ART / MENTAL HEALTH SERVICES:** |
| --- |
| You were born HIV positive and you have been on ART medication throughout your life. You have come for your routine follow up visit to the clinic.  You have had a serious boyfriend for the past 9 months. He is six years older and runs a shop near your school. A few months ago you discovered that you are pregnant and you have been having fights with your boyfriend because you feel he would have been more careful as you are still young.  Your father has become mad at you because your mother informed him that you are pregnant. From then he disowned you and vowed never to pay any school fees so you were chased out of school.  You feel like you have disappointed your family because you are the first born and they had very high hopes on you. You feel that you are too young to become a mother and are having trouble accepting the pregnancy. Your friends have left you and you just feel withdrawn, unwanted, isolated and a failure. All these have led you to not taking your medication as required. |

| **PAST MEDICAL HISTORY:** | |
| --- | --- |
| **Overall health:** | Your last visit to a health facility was about three months ago for ART refill. You are otherwise healthy, without any symptoms. |
| **Prior illness:** | You have never had any serious diseases, including TB. |
| **Prior ART use:**  **Common mental disorder screening:** | Yes  You have never been screened for any common mental disorders. Currently you feel hopeless and guilty. You have extreme mood changes of high and low and you have withdrawn from friends and activities you found fun in. |
| **STI screening:** | You have not been diagnosed with any signs and symptoms of STIs |
| **Allergies:** | None |
| **Past hospitalizations:** | Never been hospitalized or had any surgeries |

| **SEXUAL HISTORY**  You have been having sex with your serious boyfriend for the past 6 months who you met through Facebook. You had sex for the first time when you were 15 with a previous boyfriend.  You have never discussed your HIV status with your sexual partners and do not often wear condoms. | | |
| --- | --- | --- |
| **MEDICATIONS:** | | |
| **Prescription drugs:** | | You are currently on ART drugs |
| **Family planning:** | | You are not on any family planning method |
| **Over the counter (OTC) drugs, herbal or traditional medicines:** | | None |
| **Illicit/street drugs:** | | None |
| **PERSONAL HISTORY:** | | |
| **Birth date:** | 04 Sep 2004 | |
| **Birth place:** | Kiambu County | |
| **Religion:** | Christian | |
| **Alcohol:** | You occasionally take alcohol. You drink especially on weekends when you visit your serious boyfriend. | |
| **Tobacco:** | You do not smoke. | |
| **Caffeine:** | You drink a cup of tea 2-3 times per day. | |
| **Diet:** | You eat a normal diet for the area (Ugali, rice, githeri, vegetables and fruits). | |
| **Exercise:** | You exercise by walking. | |
| **Activities/hobbies/social life:** | Dancing and acting (drama club). | |
| **Stress:** | You have been having fights with your parents and serious boyfriend.  You are uncomfortable disclosing your HIV status to your boyfriend.  You are fearing to have infected your partner with HIV.  You are currently pregnant and you don’t know how to accept it.  Due to the unplanned pregnancy your father has refused to pay school fees resulting to you dropping out of school. | |
| **Occupation:** | No job | |
| **Education:** | A form 2 drop out | |
| **Living arrangement:** | You live with your parents. | |
| **Exposure to violence:** | None | |
| **FAMILY HISTORY:** | | |
| **Parents:** | Your mother is HIV positive and your father is HIV negative. | |
| **Siblings:** | You have 2 younger siblings. A brother and a sister who are healthy with no medical problems as far as you know. | |
| **Children:** | None. | |
| **Parental grandparents:** | Deceased; Grandmother died of unknown causes but was old. Grandfather died of cancer. | |
| **Maternal grandparents:** | Grandfather died in an accident. Grandmother is alive and diabetic. | |

| **AT THE END OF THE ENCOUNTER:** |
| --- |
| After the healthcare worker has completed the examination:   1. Standardized checklists will be used to evaluate the healthcare provider encounter with the SP actor. 2. The SP actor will complete checklists based on individual scenarios to provide feedback for the healthcare provider on their practice session and provide a score based on competencies met. |
