# Supplementary material for: Higher rates of mental health screening of adolescents recorded after provider training using simulated patients in a Kenyan HIV clinic: results of a pilot study
Source: Front Public Health. 2023 Sep 22;11:1209525. doi: 10.3389/fpubh.2023.1209525 (PMC10556463; doi:10.3389/fpubh.2023.1209525)
Supplement: Supplementary file 2 [file Data_Sheet_2.docx]

## **Appendix VI. Standardized Patient Training Checklist**

**Title: Developing a simulated patient encounter intervention to improve provider training around screening, counseling, and referring for common mental disorders among adolescents in an HIV clinic in Thika, Kenya**

**Protocol Version 1.2**

**Date 14 October 2020**

**Role Play ID: ____________ Actor ID: ____________ Rater ID & Date Scored: _______________________**

| **Screened for common mental disorders** | | | |
| --- | --- | --- | --- |
| **0**  **No screening done:** does not attempt to screen for common mental disorders at all | **1**  **Makes assumptions or asks wrong questions:** asks leading questions to assess for common mental disorders, assumes no mental health issues without screening, assumes mental health issues without screening | **2**  **Partially screens for mental disorders or asks indirectly about mental disorders:** completes part of a screening assessment for common mental disorders or asks indirectly about symptoms of common mental disorders (e.g., have you thought about “this” [feeling down] a lot?) | **3**  **Direct questioning and complete assessment:** conducts screening for common mental disorders in its entirety; asks questions directly using validated words or phrases (e.g., “depressed”, “hopeless”) |
| **Assessed next steps in care based on screening results** | | | |
| **0**  **No assessment done:** does not assess next steps based on screening results | **1**  **Makes incorrect assessment based on screening results:** attempts to use screening results to guide next steps in care but uses results incorrectly or makes decisions about care that do not coincide with screening results | **2**  **Assesses next steps in care with screening results but does not involve participant:** decides next steps coinciding with screening results but does not explain decision-making process with patient or involve patient in care decisions | **3**  **Correctly assesses next steps in care and involves patient:** decides next steps coinciding with screening results and involves patient actively in care decisions |
| **Problem-solving therapy: Helped client identify/prioritize problems** | | | |
| **0**  **Makes problem identification or prioritization:** does not discuss problems with the patient or help patient identify and prioritize problems | **1**  **Nonspecific discussion of problems without prioritization:** Discusses participant’s recent problems in general but does not link them to her mental health symptoms or prioritize problems. May begin counseling by talking about the patient’s problems in general or discussing many problems at once. | **2**  **Specific discussion of problems but does not prioritize them for counseling discussion:** identifies participant’s recent problems and discusses, vaguely, how these problems are related to her recent mental health symptoms. Does not attempt to prioritize problems for counseling or prioritizes problems without involving the patient. May begin counseling by talking about many of the patient’s problems at once. | **3**  **Specific discussion of problems, how they relate to mental health symptoms, and prioritizes problems:** identifies participant’s problems, specifically discusses how they relate to her symptoms of common mental disorders, and works with the patient to prioritize a problem or two for discussion during a brief counseling session. Centers the counseling session around the chosen problem(s) |
| **Problem-solving therapy: Developed action plan with client** | | | |
| **0**  **No action plan development:** does not develop an action plan with the patient around problem-solving | **1**  **Vague discussion of action plan:** discusses the basic principles of action plan development but does not guide patient in developing an action plan for her prioritized problem or apply these principles to the patient. | **2**  **Discussion of action plan but not developed with client/for prioritized problem:** develops action plan for patient but does not involve patient in the action plan development process, or does not develop the action plan for the prioritized problem of the patient’s choice. Action plan may be non-specific for the client. | **3**  **Develops specific action plan with patient tailored for the prioritized problem:** works with the patient to develop an action plan that is specific, measurable, achievable, realistic, time-bound and tailored to the prioritized problem |
| **Makes appropriate referral as needed** | | | |
| **0**  **Makes no attempt to refer:** does not attempt to refer participant but assumes they will find help on their own | **1**  **Vague referral:** Suggests a person should get help in a non-specific way (e.g., call a hotline but does not give a number, go see a counselor, doctor for recommendation) | **2**  **Specific referral or takes personal responsibility for making connection to the referral:** identifies a specific person or place by name (with a phone number), gives referral materials, or takes responsibility for making the connection between person and referral but vague and not clear (e.g., “I’ll take you to see someone”) | **3**  **Takes personal responsibility for taking action with the patient to get immediate help/connect to referral:** identifies a specific person or place by name and offers to, for example, go with the patient to a specific person or setting/office, calling together a specific person or setting/office, call parents or a friend now and go with you to talk to them |
| **Followed-up on referral as needed** | | | |
| **0**  **Makes no attempt to ask about referral or linkage to care:** does not ask patient if she successfully linked to care with the referral service and does check the patient card | **1**  **Vague follow-up:** Ask how the patient is doing and if she has spoken to anyone about her mental health needs, but does not specifically ask about whether she linked to care with the referral service | **2**  **Asks questions about referral without assessing patient satisfaction:** asks patient directly and specifically if she was successfully linked with the referral service but does not ask how the experience was, if she would go again, and if she would like any further referral | **3**  **Asks direct questions about referral and quality of care/needs for future referrals:** asks patient directly and specifically about whether she was successfully linked with the referral service; also asks about how the visit with that service was, whether she would go again, and whether she would like any additional referrals or support from the provider |
| **Communication: Active listening** | | | |
| **0**  **Unsupportive:** hostile, disconnected, or inattentive, strongly negates (3 or more negating statements)  * “I don’t think you want to do something silly/wrong/selfish like that”  * You can’t think that way | **1**  **Nonverbal support:** Eye contact, nods, facial expression of concern, open posture, leans in, does not look defensive but not much supportive language  * May make some minor negating/invalidating statements without positive/validating statements.  OR:  **Confusing, Ambivalent Support:** E.g., uses correct words with bad tone / nonverbals  * More invalidating than validating | **2**  **Supportive:** Uses an appropriate tone of voice, as well as language that encourages the person to keep talking  * Warm; demonstrates care or worry “I care about you”  ** May be empathic but undoes as in, “there are other fish in the sea” or by being self -referential | **3**  **Empathic:** emotionally responsive as demonstrated by empathic reflection - reflects feelings back accurately (only needs one solid reflection)  * Exploring patient’s experience and feelings, as in “sounds like….” “it’s really ___ when __” “that’s painful”  * Makes an attempt to express what the other person is feeling accurately  * Must be a reflection rather than an assumption. |
| **Communication: Asked clarifying questions** | | | |
| **0**  **No Questions/Comments Abrupt:** out of sync with the flow of conversation  Clearly demonstrates does not want to hear the individual’s problem and actively discourages disclosure | **1**  **Verbal Inquiries:** Encourages person to continue talking and sharing information by asking some general questions  * “tell me more about…”  * “what’s going on…” | **2**  **Asks general Qs/nonspecific in asking about mental health issues**, in a way that doesn’t really confirm/clarify  * Recent help seeking about this problem  * If they have talked to anyone else  * Seen a counselor? | **3**  **Clarifying and Confirming Questions**: Inquires and follows up to ‘decode’ the content or meaning of indirect or even direct communication  * “are you saying...”  * “what do you mean?”  * “what are you telling me?” (more than reflecting)  OR  **Asks about thoughts, feelings**  * e.g., how long have you been feeling this way?  * Warning signs (e.g., eating, sleeping, inability to focus)  * Risk factors |
