# Supplementary material for: Higher rates of mental health screening of adolescents recorded after provider training using simulated patients in a Kenyan HIV clinic: results of a pilot study
Source: Front Public Health. 2023 Sep 22;11:1209525. doi: 10.3389/fpubh.2023.1209525 (PMC10556463; doi:10.3389/fpubh.2023.1209525)
Supplement: Supplementary file 3 [file Data_Sheet_3.docx]

Tests for autoregression suggested a possible first order autoregressive – AR(1) – process. ARIMA models using an AR(1) process resulted in a smaller but significant effect size of CMD screening proportion immediately post-SPE training (RR: 1.22, 95% CI: 1.04 – 1.43, p<0.01). We found similar trends to the primary model across other sensitivity analyses. Truncating the week immediately post intervention resulted in a smaller magnitude of effect size of the SPE intervention on the immediate change in CMD screening proportion (RR: 2.00, 95% CI: 1.01-3.94, p=0.05). Sensitivity analyses with aggregation by day resulted in a significant but slightly smaller effect size of the SPE intervention on the immediate change in CMD screening proportion (RR: 2.43, 95% CI: 1.29-4.61, p<0.01) and aggregation by month resulted in a larger magnitude of effect size (RR: 3.16, 95% CI: 1.65-6.08, p<0.01).
